# Adaptive radiotherapy for head and neck cancer: Pitfalls and possibilities from the radiation oncologist's point of view

**DOI:** 10.1002/cam4.7192

**Published:** 2024-04-23

**Authors:** Sandra Nuyts, Heleen Bollen, Avrahram Eisbruch, Primoz Strojan, William M. Mendenhall, Sweet Ping Ng, Alfio Ferlito

**Affiliations:** ^1^ Laboratory of Experimental Radiotherapy, Department of Oncology KU Leuven Leuven Belgium; ^2^ Department of Radiation Oncology Leuven Cancer Institute, University Hospitals Leuven Leuven Belgium; ^3^ Department of Radiation Oncology University of Michigan Ann Arbor Michigan USA; ^4^ Department of Radiation Oncology Institute of Oncology University of Ljubljana Ljubljana Slovenia; ^5^ Department of Radiation Oncology University of Florida College of Medicine Gainesville Florida USA; ^6^ Department of Radiation Oncology Olivia Newton‐John Cancer and Wellness Centre, Austin Health Melbourne Australia; ^7^ Coordinator International Head and Neck Scientific Group Udine Italy

**Keywords:** adaptive radiotherapy, head and neck cancer, review

## Abstract

**Background:**

Patients with head and neck cancer (HNC) may experience substantial anatomical changes during the course of radiotherapy treatment. The implementation of adaptive radiotherapy (ART) proves effective in managing the consequent impact on the planned dose distribution.

**Methods:**

This narrative literature review comprehensively discusses the diverse strategies of ART in HNC and the documented dosimetric and clinical advantages associated with these approaches, while also addressing the current challenges for integration of ART into clinical practice.

**Results and Conclusion:**

Although based on mainly non‐randomized and retrospective trials, there is accumulating evidence that ART has the potential to reduce toxicity and improve quality of life and tumor control in HNC patients treated with RT. However, several questions remain regarding accurate patient selection, the ideal frequency and timing of replanning, and the appropriate way for image registration and dose calculation. Well‐designed randomized prospective trials, with a predetermined protocol for both image registration and dose summation, are urgently needed to further investigate the dosimetric and clinical benefits of ART.

## 1 INTRODUCTION

Head and neck cancer (HNC) is the seventh most common cancer and cause of cancer‐related death worldwide with more than 500,000 new cases and 380,000 deaths yearly.^1^ HNC is typically diagnosed in a locally advanced yet potentially curable stage, for which radiotherapy (RT), either alone or in combination with concurrent chemotherapy, is usually the treatment of choice.^2^, ^3^ Administering high‐dose RT to the tumor, referred to as the “target volume” (TV), is essential for achieving curation.^4^ Over the last decade, the technological advancement from 3D RT to more conformal techniques such as IMRT and VMAT have dramatically improved the precision of RT delivery. Patients with HNC often experience anatomical changes during the course of RT, which may result in unintended dosimetric changes affecting the side effect profile and potentially the efficacy of the treatment.^5^ With the general introduction of IMRT and VMAT in most RT centers worldwide, the consequences of anatomical changes that may occur during treatment may be more dramatic than in conventional treatments because of the sharp dose gradients between the edges of the TVs and the critical organs at risk (OAR). Adaptive RT (ART) involves obtaining a new set of images during treatment and adjusting the parameters of the RT plan in response to the updated findings. The new RT plan will then be used for the remainder of the treatment, with the intent of increasing the precision of TV coverage and reducing overdosage to the OAR. Although ART is an appealing concept, the clinical impact of this approach on both long‐term treatment toxicity and tumor control for HNC patients remains unclear. Furthermore, its integration into clinical practice is currently hampered by its labor intensity and cost. In addition, several questions exist regarding accurate patient selection and the appropriate timing regimen. ART can be initiated either in reaction to a specific trigger, such as weight loss or at predetermined intervals throughout the course of the treatment. Ongoing efforts aim to identify a dependable method for triggering re‐CT and/or replanning in HNC patients. With the increasing number of indications for proton therapy for HNC and because of the emphasized precision of the proton beam, addressing these challenges is becoming increasingly urgent.^6^ This narrative literature review discusses the diverse strategies of ART in HNC and the documented dosimetric and clinical advantages associated with these approaches, while also addressing the current challenges for integration of ART into clinical practice.

## 2 ANATOMICAL CHANGES DURING RADIOTHERAPY AND THEIR DOSIMETRIC CONSEQUENCES

Numerous patients may undergo volumetric and spatial alterations in the TVs and OAR during treatment, resulting from a combination of factors such as tumor response, weight loss, post‐operative changes, inflammation, and the impact of radiation on normal tissues. These changes have the potential to introduce significant uncertainty regarding the actual dose received by the TVs and OAR, as these variations are not considered in the initial planning.^7^ Weight loss, estimated to occur in almost 80% of the patients, is a well‐known problem in patients treated with (chemo)RT for HNC.^8^, ^9^ The median weight change reported during IMRT for HNC ranges between 6.5% and 10%, although large variations have been described (+5.6% to −24.7%).^10^, ^11^, ^12^, ^13^, ^14^, ^15^ Several trials have identified weight loss in HNC patients as an important risk factor for geometric shifts of TVs and OAR during treatment and as a main indication for replanning.^16^, ^17^, ^18^, ^19^

There is a widely acknowledged concern that TVs may experience dose inhomogeneities, creating cold spots that have been associated with worse local control in non‐randomized cohorts.^20^, ^21^ If ART could address these inhomogeneities, the occurrence of cold spots could be minimized, potentially leading to a reduction of in‐field failure rates. The observed reduction in tumor size during treatment exhibits considerable heterogeneity across studies, likely attributable to the diverse spectrum of radio‐responsiveness among the different HNC subtypes.^22^, ^23^ Furthermore, the definition of the TV reported is variable within trials, as some studies report the gross tumor volume (GTV), others the high‐risk clinical TV (CTV) with variable margins, and others the high‐risk planning TV (PTV), making comparisons across studies challenging. Detectable decreases in tumor size can manifest as early as the first 2 weeks, with the median shrinkage ranging between 3% and 16% by the end of weeks 2% and 7% to 48% by the end of weeks 4% and 6% to 66% by the end of the treatment.^5^, ^10^, ^15^, ^22^, ^23^, ^24^, ^25^, ^26^, ^27^, ^28^, ^29^, ^30^, ^31^, ^32^, ^33^, ^34^, ^35^, ^36^, ^37^, ^38^, ^39^, ^40^, ^41^ As to involved nodes, similar broad ranges of shrinkage as the primary tumor are reported.^23^, ^24^, ^28^, ^29^, ^36^, ^37^, ^38^, ^41^, ^42^ Barker et al. conducted a prospective trial, examining anatomical changes in TVs on 222 CT scans three times a week during the course of the treatment. The GTV of the primary tumor (GTVp) exhibited a median decrease of 1.8% per treatment day, resulting in a median final GTVp shrinkage of 69.5%.^43^ Additionally, the center of mass of the GTVp showed a change in position over time, suggesting that tumor loss often occurred asymmetrically. Upon treatment completion, the median displacement of the center of mass amounted to 3.3 mm. In a prospective feasibility study conducted at MD Anderson Cancer Center, five patients diagnosed with locally advanced HPV‐positive oropharyngeal carcinoma (OPC) underwent definitive chemoRT, accompanied by intra‐treatment MRI every 2 weeks. The GTVp demonstrated an average reduction of 44%, 90%, and 100% at weeks 2, 4, and 6, respectively, with corresponding nodal volumes decreasing by 25%, 60%, and 80%.^44^ Similarly, in a related study, Hamming‐Vrieze et al. observed a 70% average reduction in GTVp on MRI imaging by week 6 in patients with OPC.^45^ The impact of changes in TV on dosimetry is less investigated. Conflicting results are found when planned and delivered doses without ART are compared for GTV, CTV, and PTV. While most studies report very low dose differences (<1%–2% for the D2% and D95%), some (less recent) trials found significant dose differences such as a 2 Gy reduction of the minimum dose (Dmin) to the PTV.^10^, ^46^, ^47^

Regarding OAR, the parotid glands (PG) are recognized to undergo shrinkage throughout the treatment course. This process may begin as early as the first 2 weeks and exhibits significant heterogeneity among HNC patients. In addition to the volumetric change, the PGs exhibit a distinct pattern of superior and medial translocation during RT, likely attributed to the shrinkage of the TV and the patient's weight loss.^17^, ^18^, ^25^, ^48^ This migration may unintentionally result in overdosage of the PGs, a factor associated with more pronounced xerostomia as observed in small retrospective trials.^10^ Table 1 provides an overview of the existing studies investigating volumetric and dosimetric changes for the PGs during curative IMRT for HNC. All 29 included studies confirm an average decrease in parotid volume, resulting in an increase in the mean dose (Dmean). The difference between the Dmean that was initially planned and the actual administered Dmean ranges from 0.9 to 5 Gy. Weight loss, initial BMI, Dmean on the PG in the initial RT planning, and initial PG volume were associated with PG volume loss and increased Dmean on the PGs during treatment.^12^, ^17^, ^18^, ^19^, ^52^, ^61^ Notably, the increase in PG dose seemed to be substantially patient‐dependent and was observed in only 30%–65% of patients. Thus, the assessment of the median and mean dose increase on the PGs in HNC patients is likely an underestimation. A selected number of patients will likely experience a more pronounced and clinically relevant increase in the mean PG dose. O'Daniel et al. reported a mean increase of 3 Gy on the ipsilateral PG during the treatment course. However, in 45% of included patients, an increase of up to 7 Gy was seen.^49^

**TABLE 1:** Studies reporting volumetric, positional and dosimetric changes of the parotid glands during curative IMRT for HNC.

References	Sample size	Timing re‐scan	Dose calculation	Volume analysis	Positional analysis	Changes in dosimetry
Hansen et al. (2006)^48^	13	After mean delivered dose of 38 Gy	Rigid	Mean % change: ↓ 21.5% left ^ICNS^ ↓ 15.6% right ^ICNS^	N.A	*V*_26 Gy_↑ 11% ^ICNS^ *D*_mean_ ↑ 1 Gy ^ICNS^
O'Daniel et al. (2007)^49^	11	Twice/week	DIR	N.A	N.A	*D*_mean_^ipsi^ ↑ 3 Gy *D*_mean_^contra^ ↑ 1 Gy
Robar et al. (2007)^50^	15	Weekly	DVH	Mean ↓ 4.9%/week ^ICNS^	Mean medial translocation: 0.85 mm/week	Left ^ICNS^: *D*_mean_↑ 2.6% ± 4.3%, *V*_26 Gy_ ↑3.5% ± 5.2% Right ^ICNS^: *D*_mean_ ↑ 0.2% ± 4.0% *V*_26 Gy_ ↑ 0.3% ± 4.7%
Han et al. (2008)^51^	5	Daily (MVCT)	Rigid	Mean ↓ 1.1%/day ^NS^	N.A	*D*_median_ ↑ 0.17 Gy/day ^NS^
Vasquez et al. (2008)^52^	10	After mean delivered dose of 46 Gy	DIR	Mean % change: ↓ 17% ± 7% ^ipsi^ ↓ 5% ± 4% ^contra^	Mean medial and posterior translocation: 3 mm	N.A
Lee et al (2008)^17^	10	Daily (MVCT)	DIR	N.A	N.A	*D*_mean_↑ 15% ^NS^ *D*_mean_↑ 3 Gy
Bhide et al. (2010)^10^	20	Weekly	DIR	Mean % change: week 2: ↓14.7% ^NS^ End RT: ↓35% ^NS^	Mean medial translocation week 4: 2.3 mm	week 4: *D*_mean_^ipsi^ ↑ 2.8 Gy
Height et al. (2010)^53^	10	After mean delivered dose of 40–50 Gy	DVH	Mean ↓ 23.5% ^NS^	Minimal medial translocation	Minimal
Ahn et al. (2011)^47^	23	Fx 11, 22 and 33	DVH	Mean % change: ↓ 24% right ^ICNS^ ↓ 28% left ^ICNS^	N.A	*D*_mean_ ↑ 2.5 Gy ^NS^
Beltran et al. (2012)^32^	16	Fx 15 and 25	DVH	Mean ↓ 30% ^NS^	N.A	*D*_mean_^ipsi^ ↑ 4.7% *D*_mean_^contra^ ↑ 6.1%
Lu et al. (2012)^29^	43	Fx 20	N.A.	Mean % change: ↓ 35.5% left ^ICNS^ ↓ 36.8% right ^ICNS^	N.A	N.A
Fiorentino et al. (2012)^54^	10	Daily	N.A.	Mean % change: ↓ 43.5% ^ipsi^ ↓ 44.0% ^contra^	N.A	N.A
Ho et al. (2012)^55^	10	Weekly	DVH	Mean % change: ↓ 29.7% ^ipsi^ ↓ 28.4% ^contra^	N.A	*D*_mean_ ↑ 1.1 Gy ^NS^
Jensen et al. (2012)^15^	72	Weekly	DIR	N.A	N.A	*D*_mean_^ipsi^ ↑ 4% *D*_mean_^contra^ ↑ 1%
Marzi et al. (2012)^56^	15	Weekly	DVH	N.A	N.A	*D*_mean_ ↑ 1.3 Gy ^NS^ *D*_mean_ ↑ 0.22 Gy/week
Schwartz et al. (2013)^35^	22	Weekly	DIR	Mean ↓ 26% ^NS^	N.A	*D*_mean_^ipsi^ ↑ 0.4 Gy ^NS^
Nishi et al. (2013)^57^	20	3th or 4th week	DVH	Mean 81.9 % ^NS^	Mean medial translocation: 4.2 mm	*D*_mean_ ↑ 5.0 Gy ^NS^
Hunter et al. (2013)^11^	18	Weekly	DVH	N.A	N.A	*D*_mean_ ↑ 2.2 Gy ^NS^ (63% of PGs)
Berwouts et al. (2013)^58^	10	Fx 8 and 18	DIR	N.A	N.A	*D*_mean_^ipsi^ ↑ 1.6 Gy *D*_mean_^contra^ ↑ 0.9 Gy
Hunter et al. (2013)^11^	18	Weekly	DIR	Mean ↓ 26% ^NS^	N.A	*D*_mean_ ↑ 2.2 Gy ^NS^
Sanguineti et al. (2013)^12^	85	Weekly	N.A.	Mean ↓ 31%	N.A	*D*_mean_ ↑ 0.4 – 27.9 Gy
Jin et al. (2013)^34^	10	Fx 23	Rigid	Mean ↓ 39% (8.2 cc) ^ICNS^	N.A	N.A
Huang et al. (2015)^37^	19	Weekly	DIR	Mean % change: Fx 20: ↓ 15.1% End RT: ↓ 38.0% ± 15.3% ^ICNS^	Mean medial translocation: 3.1 mm	*D*_mean_ ↑ 4.4 Gy ^ICNS^
Castelli et al. (2015)^22^	15	Weekly	DIR	Mean ↓ 28.3% ^NS^	N.A	*D*_mean_↑ 3.7 Gy ^NS^ (59% of PGs)
Yao et al. (2015)^18^	50	Weekly	DIR	Mean ↓ 35% ^NS^ (6.8%–69.4%)	N.A	*D*_mean_↑ 3.52 Gy (11.4%) ^NS^
Zhang et al. (2016)^39^	13	Weekly	DIR	Mean % change: ↓ 34.5 % ^ipsi^ (10%–57.6%) ↓ 28 % ^contra^ (−5.2% to 57.3%)	N.A	*D*_mean_↑ 4.1 Gy
Mahmoud et al. (2017)^30^	22	Fx 15, 27	Rigid	Mean ↓ 31% ^NS^	N.A	*D*_mean_↑ 16% ^NS^
Zhang et al. (2017)^59^	39	Fx 10, 20, 30	DIR	Mean % change: ↓ 37% ^ipsi^ ↓ 35% ^contra^	Mean medial translocation: 3 mm	N.A
Hu et al. (2018)^60^	40	Fx 22	DIR	Mean % change: ↓ 17.2 % ^ipsi^ ↓ 20 % ^contra^	N.A	N.A

*Note*: For studies encompassing a broad spectrum of reported fractions at the time of re‐scan, the volumetric and dosimetric change and anatomical shift of the PGs of the last re‐scan were noted. The registration method for imaging and dose calculation is given (rigid registration, DIR, or calculation of DVH on synthetic CT).

Abbreviations: ^contra^, contralateral; DIR, deformable image registration; DVH, dose volume histograms (on synthetic CT); fx, fraction; Gy, Gray; ^ICNS^, parotid side (left or right) was specified but ipsilateral and contralateral designation were not explicitly stated; ^ipsi^, ipsilateral; mm, millimeter; MVCT, megavoltage computed tomography (MVCT); N.A, not available or applicable; ^NS^, parotid side was not specified; PG, parotid gland.

Less consistent findings exist regarding volume/position and dosimetric changes for the spinal cord and the brainstem. Several small studies report a significant increase in the maximal dose (Dmax), due to hot spots developing during the treatment course,^10^, ^25^, ^26^, ^33^, ^37^, ^48^, ^62^ while other studies report no dosimetric change at all.^34^, ^49^, ^60^, ^63^, ^64^ ART with replanning is frequently applied in clinical practice when the pre‐defined thresholds for the spinal cord or brainstem are exceeded, although noteworthy deviations with clinical significance may manifest in only a minority of patients.^24^, ^37^ In a replanning study on 13 patients, Hansen et al. found the Dmax of the spinal cord to be increased in all patients, while 85% of patients showed an increase in Dmax of the brainstem.^48^ Most patients, however, only show a modest increase in the spinal cord Dmax of 2–4 Gy, although increases up to 15.4 Gy for the spinal cord and 8.1 Gy for the brainstem have been described.^26^, ^37^, ^48^ In most cases, however, the increase was not considered clinically significant, as the recalculated Dmax did not exceed the hard, universally accepted, spinal dose constraint of 45–48 Gy.^65^

Beltran et al. found an increase in the mean oral cavity dose of 3.5% at 3 weeks after the start of RT in 16 patients.^32^ Hansen et al. documented an average increase in the Dmax of the mandible and the percentage of the mandible volume receiving ≥60 Gy (*V*_60_) and 70 Gy (*V*_70_) without replanning. However, variations in *V*_70_ were not proven to be statistically significant.^48^ A significant volume loss of 20% ± 10% for the ipsilateral submandibular gland (SMG) and 11% ± 7% for the contralateral SMG was described by Vasquez et al. In addition, a cranial, medial, and posterior shift was described for irradiated SMGs, while the spared SMGs showed only a small deformation.^52^

A limitation of studies investigating volumetric and consequent dosimetric changes during treatment is the difficulty in separating influences over dose distribution due to rigid errors (i.e., inter‐fractional setup and intra‐fractional variations) and non‐rigid anatomical changes during the RT course. As shown in Table 1, image registration protocols differ between studies, and as discrepancies from the initial plan are sometimes minimal, the interpretation of the results must be approached with caution.

## 3 EVIDENCE FOR ADAPTIVE REPLANNING: BENEFITS OF DOSIMETRY

While it is clear from the previously mentioned results that alterations in the patient's anatomy during treatment may potentially lead to a higher actually delivered dose than initially anticipated, the actual benefits of making a new RT plan during treatment are less investigated. Table 2 includes a total of 13 studies investigating the dosimetric impact of ART on both OAR and TVs. In a prospective study involving 22 patients with OPC, Schwartz et al. assessed the influence of replanning using daily CT.^35^ All 22 patients had at least 1 adaptive replan and 8 patients underwent 2 replans. In the dosimetric analysis, patients who underwent one replanning exhibited a reduction of 1.3 Gy (*p* = 0.002) in Dmean to the ipsilateral parotid gland (PG) and 0.6 Gy (*p* = 0.003) to the contralateral PG. The second replanning resulted in a further decrease in Dmean to the ipsilateral and contralateral PG by 4.1 and 0.8 Gy, respectively. These concerned the static dose differences since no cumulative dose summation was performed. Additionally, reduced doses to the oral cavity and larynx were observed. Duma et al., while not being able to objectify a dosimetrical benefit for the PGs, described a significant improvement in the dose to the oral cavity, larynx, and spinal cord with ART.^66^ Castelli et al. documented that through replanning, the average reduction in the mean parotid dose was 5.1 Gy (with a maximum of 12.2 Gy), and the absolute risk of xerostomia was lowered by 11% (with a maximum reduction of 30%). Notably, the ART strategy seemed advantageous not only for patients with objectively over‐irradiated PGs but also for those who did not exhibit an increased dose of the PGs before the initiation of replanning.^22^

**TABLE 2:** Dosimetric benefits of ART in HNC patients treated with IMRT: results of replanning studies.

References	Sample size	Tumor site	Timing re‐scan	Method to cumulate dose	PG (Dmean)	Spinal cord (Dmax)	PTV
Zhao et al. (2011)^63^	33	NPC	Fx 15	DVH	−0.1 Gy ^ipsi^ −0.35Gy* ^contra^	↓Dmax*	V95 ART: 97%* No ART:85%*
Ahn et al. (2011)^47^	23	Various	Fx 11, 22 and 33	DVH	+ 2.5 Gy*	+ 3.8 Gy*	D99 ↑ 3.9 Gy*
Capelle et al. (2012)^25^	20	Various	3rd week	DVH	−0.6 Gy* ^NS^ (−1.2 Gy for NPC only)	−0.5 Gy* (−1.2 Gy for NPC only)	↑ coverage 0.5 Gy (D1%)*
Duma et al. (2012)^66^	11	Various	Fx 16	DVH	No benefit	−0.14 Gy*	N.A
Jensen et al. (2012)^15^	15	Various	Weekly	DIR	−3.8% ^ipsi^ −11.5% ^contra^	N.A	↑ coverage 8%
Schwartz et al. (2013)^35^	22 (8 pts with 2 ART replans)	OPC	Fx 15 and 22 (if 2 replans)	DIR	1 ART replan: −1.3 Gy* ^ipsi^ (4%) 2 ART replan: −4.1 Gy* ^ipsi^ (9%)	N.A	↑ coverage and dose homogeneity
Nishi et al. (2013)^57^	20	Various	Fx 21–28	DVH	−5.3 Gy* ^NS^	−2.1 Gy*	D98 ↑ 0.8 Gy*
Olteanu et al. (2014)^67^	10	Various	Fx 8 and 18	DIR	−6%* ^NS^ (median)	N.A	↑ Dmin ↓ Dmax
Chitapanarux et al. (2015)^26^	17	NPC	Fx 17	DVH	−0.9 Gy ^ipsi^ −1.1 Gy* ^contra^	−3.75 Gy*	D95 ↑ 0.2‐2.6 Gy
Castelli et al. (2015)^22^	15	LAHNC	Weekly	DIR	−5.1 Gy* ^NS^	N.A	N.A
Dewan et al. (2016)^27^	30	Various	Fx 20	DVH	−6 Gy* ^ipsi^ −2.2 Gy* ^contra^	−6 Gy*	More uniform coverage V110% ↓ by 2*
Zhang et al. (2016)^39^	13	OPC	Weekly	DIR	3 ART replans: −3.1 Gy* ^NS^ 6 ART replans: −3.3 Gy* ^NS^	N.A	N.A
Hu et al. (2018)^60^	40	NPC	Fx 22	DIR	−0.7 Gy* ^ipsi^ −0.1 Gy ^contra^	−0.4Gy	D95 ↑ 13%*
Maheshwari et al. (2020)^68^	60	LAHNC	Fx 16–20	DIR	−4 Gy (6%) ^ipsi^ −1.9 Gy (2.2%) ^contra^	−3 Gy (4.3%)	—

*Note*: The method for dose accumulation is given (DIR or calculation of DVH on synthetic CT).

Abbreviations: ^contra^, contralateral; DIR, deformable image registration; DVH, dose–volume histogram; fx, fraction; Gy, Gray; ^ipsi^, ipsilateral; LAHNC, locally‐advanced HNC; N.A, not available or applicable; NPC, nasopharyngeal carcinoma; ^NS^, parotid side was not explicitly stated; OPC, oropharyngeal carcinoma.

* *p* < 0.05.

As shown in Table 2, ART facilitated a reduction in PG dose, ranging from 0.6 to 4.1 Gy across all studies, except one that did not observe a benefit with a single replanning session.^66^ This finding is of notable significance because of the well‐established radio‐sensitivity of the PGs. Lower parotid doses are known to result in reduced severe xerostomia and better salivary flow although the relationship between RT dose and patient‐reported xerostomia is complex.^69^, ^70^, ^71^, ^72^, ^73^, ^74^ Regarding the spinal cord, eight trials reported a dosimetric advantage, resulting in a decrease of the Dmax by 0.1–6 Gy over multiple fractions.^25^, ^26^, ^27^, ^57^, ^60^, ^63^, ^66^, ^68^ The clinical relevance of these dosimetric differences remains uncertain. No data exist on the benefit of ART on other important OAR, e.g. the oral cavity, SMGs, or pharyngeal constrictor muscles. Concerning the dosimetric benefits of ART on the PTV, all studies in Table 2 report more uniform coverage^27^, ^35^, ^67^ and/or increased coverage.^15^, ^25^, ^35^, ^48^, ^60^, ^63^ The results of the studies mentioned in Table 2 thus favor the adaptive approach, but also reveal considerable heterogeneity in patient‐specific benefit.

The primary limitation in studies exploring the dosimetric advantages of ART lies in the ongoing investigation of universally accepted recommendations and methods for dose summation. Dose comparisons can be conducted either at the individual fraction level or by considering the accumulated dose, which can be calculated based on the Dmean for a subset of fractions or the entire treatment. Discrepancies in fraction doses resulting from adaptations may not necessarily correlate with meaningful changes in clinical parameters. To assess clinical relevance, information about the cumulated dose is imperative. Enhanced fraction doses hold greater significance if the summed dose falls within the steep region of the dose–response curve.^67^ Dose accumulation can be estimated using either rigid or non‐rigid, thus deformable, Image Registration (DIR), as is the case for several studies (Table 2). However, a review of the literature reveals that DIR is not yet validated for routine clinical practice.^75^ Another approach involves generating hybrid plans by registering the initial plan to the repeat CT to evaluate dose coverage for TVs and OAR, comparing dose–volume histograms (DVH). Furthermore, it is evident that the benefits of ART are more apparent when assessing dosimetry on an individual patient basis, rather than through population averages. It must also be recognized that patients who underwent ART constitute a heterogeneous group, as they were replanned at different time points for various reasons, using different platforms (i.e. KV CT vs. MV CT), and had diverse tumor locations and volumes. The reported effects on OAR and TVs are thus influenced by many variables.

## 4 EVIDENCE FOR ADAPTIVE REPLANNING: CLINICAL OUTCOMES

While the overall influence of replanning on dosimetric alterations is extensively documented, it remains uncertain whether the dosimetric enhancements achieved with ART translate into meaningful clinical benefits. The French ARTIX trial is the only phase 3 randomized clinical trial comparing weekly ART with standard IMRT in 132 patients with locally advanced OPC.^76^ The aim of this study was to improve salivary gland function, with salivary quantification as primary end point and secondary end points including salivary gland excretory function by scintigraphy, patient‐reported outcomes, early and late toxic effects, and cancer‐specific outcomes. Despite a maximal and labor‐intensive approach, the primary end point of decreased xerostomia was not reached. Patient‐reported xerostomia did not differ between the arms. The only measured outcome that differed between both groups was the mean parotid excretory function as estimated by scintigraphy, in which patients treated with ART showed a benefit. The authors highlight the need to identify the subset of patients who may benefit from a personalized ART strategy. Eight other studies investigated clinical outcomes after ART, as summarized in Table 3. Acute and late toxicity scores are reported, as well as loco‐regional control (LRC) and overall survival (OS) rates. Four studies had a prospective study design,^57^, ^68^, ^77^, ^78^ of which one was randomized.^68^ The remaining trials were retrospective,^20^, ^21^, ^38^, ^63^ of which two studies used case‐matched cohorts.^20^, ^63^ The study populations varied in size, ranging from 20 to 317 patients across different studies, totaling 883 patients. The median follow‐up time ranged from 6 to 70 months. The predominant tumor locations were the oropharynx and nasopharynx. Seven trials exclusively enrolled patients treated with definitive (chemo) RT, while one study also included patients who had undergone surgery prior to RT.^21^ PTV was created by using a 3–5 mm margin around the CTV. The replanning was executed either at specific time points^38^, ^57^, ^68^, ^78^ or at the discretion of the radiation oncologist (RO).^20^, ^21^, ^63^, ^77^

**TABLE 3:** Clinical benefits of ART in HNC patients treated with IMRT: results of replanning studies.

References	Sample size		Tumor site	Total dose to PTV	Timing	Follow‐up (months)	Clinical endpoint		
	ART	No ART					Acute toxicity	Late toxicity	LRC/OS
Castelli et al. (2023)^a^ , ^76^	67	65	OPC	70	Weekly	26	No difference in G2 and G3 mucositis, xerostomia, leukopenia, anemia, dysgeusia, or oropharyngeal pain	No difference in xerostomia at 12 months Mean stimulated salivary flow same both arms	2 years LRC: 76.3% ART 77.5% no ART 2 years OS 76.9% both arms
Zhao et al. (2011)^63^	33	66	NPC	66–70	Av Fx 15 (±5)	38	/	↓ xerostomia and mucositis with ART for N2‐3 pts*	3 years LRFS: ART: 72.7%* No ART: 68.1%* Effect most pronounced in AJCC stage ≥ T3 and in case of large lymph nodes
Schwartz et al. (2012)^b^,^77^	22	0	LA OPC	66‐70	1ART plan: Av Fx 16 2ART plans: Av fx 11 and 22	31	G2 and G3 mucositis: 100% G2/G3 xerostomia: 55%/5%	Maintenance or recovery of speech/eating abilities at 20 months	2 years LRC: 95.0%
Yang et al. (2013)^b^,^78^	86	43	NPC	70–76	Fx 15 and/or 25	29	↑ QOL in ART‐arm*	↑ QOL in ART‐arm*	2 years LRC/2 years OS: ART: 97.2%*/89.8% No ART: 82.2%*/82.2%
Nishi et al. (2013)^b^,^57^	20	0	Various	70	Fx 21–28	57	/	No ≥ grade 2 xerostomia	2 years LRC: 100% (NPC), 50.0% (OPC, HPC)
Chen et al. (2014)^21^	51	266	LA HNC	60–70	At median of 40 Gy	30	G3 toxicity: ART: 39% No ART: 30%	G3 toxicity: ART: 14% No ART: 19% No significant difference in gastrostomy tube dependence at 1 year	2 years LRC/2 years OS: ART: 88.0%*/73.0% No ART: 79.0%*/79.0%
Kataria et al. (2016)^38^	36	0	LA HNC	66–70	Fx 23	31	G2‐3 mucositis: 100%	G2 xerostomia: 8% G2 mucositis: 11% No G3 toxicity	2 years DFS: 72.0% 2 years OS: 75.0%
Luo et al. (2016)^20^	66	66	NPC	66–76	At median of 44 Gy	70	/	/	5 years LRC/OS: ART: 96.7%*/72.6% No ART: 88.1%*/69.0%
Maheshwari et al. (2020)^a^ ^,^ ^b^,^68^	30	30	LA HNC	70	Fx 16–20	6	G2 toxicity: no significant differences G3 xerostomia: ART: 30%* No ART: 50%*		LRC after 6 months: ART: 96.7% No ART: 90%

*Note*: /, information not available or applicable.

Abbreviations: AJCC, American Joint Committee on Cancer; Av, Average; Fx, fraction; G2, grade 2; G3, grade 3; HNC, head and neck cancer; LA, locally advanced; LRC, locoregional control; LRFS, loco‐regional free survival; NPC, nasopharyngeal carcinoma; OPC, oropharyngeal carcinoma; OS, overall survival; QOL, quality of life.

^a^ Randomized study.

^b^ Prospective studies, non‐randomized.

* *p* < 0.05.

A large prospective, non‐randomized trial on adaptive replanning was performed on patients with nasopharyngeal carcinoma (NPC) by Yang et al. Eighty‐six of 129 patients received a replanning. Two‐year LRC was significantly better in the ART group and various quality‐of‐life parameters showed significant improvement.^78^ In a large retrospective trial, Chen et al. compared disease control outcomes of 317 patients with locally advanced HNC, of which 16% received an adaptive replan. The two‐year LRC was superior in the ART group (88% vs. 79%, *p* = 0.01), with all failures observed within the high‐dose PTV.^21^ In a propensity score‐matched analysis, 66 patients undergoing definitive CRT for T3/T4 NPC were paired with 66 patients who did not undergo replanning. ART significantly enhanced the 3‐year locoregional free survival in patients with stage T3‐T4 only. In cases of early‐stage disease (T1‐T2) or with large lymph node volumes (N2, N3), there was no notable distinction between the two groups. Nevertheless, the predominant pattern of failure, distant metastases, exhibited no significant disparity between the ART and non‐ART cohorts.^63^

As shown in Table 3, the reported 2‐year OS among studies ranged from 73% to 90%, which is consistent with findings reported in the literature for patients who did not undergo ART.^69^, ^79^ Studies incorporating a comparison arm did not reveal a significant difference in OS between the two approaches (ART or no ART).^21^, ^78^ Regarding improvement in toxicity, acute and late toxicity rates were comparable with previously published toxicity rates for patients treated without ART.^69^, ^72^ In the trial of Schwartz et al., the quality of life score at 20 months indicated either complete preservation or the functional recovery of speech and eating. Nonetheless, the authors emphasized that these results require validation in a larger cohort of patients.^77^ In the three studies featuring a comparison arm, Chen et al.'s trial revealed no significant difference in late toxicity between the cohorts.^21^ Zhao et al. described a significant decrease of xerostomia and mucositis in the ART arm, although exclusively observed in patients with N2,3 NPC.^63^ The study by Yang et al. showed a significant improvement in the score on the EORTC QLQ‐C30 quality of life questionnaire. Furthermore, significant differences were noted in the occurrence of appetite loss, speech problems, dry mouth, and sticky saliva, opening the mouth, and social functioning.^78^

In summary, the limited retrospective and non‐randomized prospective trials available present promising outcomes, with several studies indicating improvement of LRC and reduced toxicity in patients undergoing replanning compared to control patients. Additionally, one study reported a significant improvement in quality of life. However, the absence of randomized trials compromises the robustness of the evidence. Furthermore, in most studies, replanning was initiated based on the RO's individual opinion. Since tumor shrinkage was indicated as one of the main reasons for carrying out ART, the potential benefit observed in LRC may be explained by the selection of tumors that exhibit a higher sensitivity to RT, possibly indicating more favorable tumor biology. In addition, there was a predominance of tumors located in the nasopharynx and, to a lesser extent, the oropharynx. Furthermore, given the non‐randomized trial design of the study by Yang et al., the impact of ART on quality‐of‐life measures could be influenced by confounding factors, e.g. baseline disparities, replanning at various time points, and variations in tumor location and volume. However, under the condition that ART is performed carefully, it can be concluded that replanning does not compromise disease control in a definitive RT setting. Nevertheless, there is an immediate need for well‐designed randomized prospective trials, with a predetermined protocol for both image registration and dose summation, to confirm the clinical relevance of ART. Furthermore, existing studies almost exclusively focus on the reduction of xerostomia, while possibly insufficient attention is given to dysphagia, another frequent side effect with major implications on the patient's quality of life. In a comparison between planned dose, delivered doses, and patient‐reported outcomes, Weppler et al. found that dysphagia‐focused ART may provide the greatest toxicity benefit for HNC patients regarding patient‐reported outcomes.^80^ More attention to reducing the dosage of swallowing muscles in the adaptive process could, therefore, underline its clinical benefits.

Regarding the postoperative setting, the impact of ART on toxicity and clinical outcomes is not clear, given the scarcity of data. Capelle et al. were unable to establish the advantage of ART for post‐operative HNC patients,^25^ while Chen et al. emphasized the importance of ART in an adjuvant setting considering the known swelling of the surgical graft and wound site.^21^ In a small, prospective trial on 22 patients with HNC, dose recalculation was performed at weeks 1, 3, and 6 to determine the need for ART. In the adjuvant setting, a replanning was considered necessary in only 18% of patients, compared to 73% in the definitive setting. The authors explained this significant difference by the fact that patients receiving definitive RT experienced significantly more weight loss and shrinkage of the high‐risk CTV.^30^

## 5 PITFALLS FOR CLINICAL IMPLEMENTATION

### 5.1 Patient selection for ART

While numerous trials have indicated a general trend towards decreased doses to OAR and enhanced TV coverage with the use of ART, the precise method for identifying patients who would gain maximum benefit from replanning remains uncertain. There is a large variability in the literature regarding the proportion of patients that may experience advantages from ART. Ahn et al. reported a dosimetric benefit of replanning for 65% of patients, while Schwartz et al., Chen et al., and Figen et al. found a benefit in 35%, 16%, and 10.6% of patients, respectively.^21^, ^35^, ^47^ Since selection criteria are currently poorly defined, most studies select patients for ART at the discretion of the RO, which translates into a low percentage of patients undergoing ART.^21^ Considering the substantial variability in anatomic changes affecting both TVs and OAR, one singular ART regimen may not be universally applicable to all HNC patients. Ideally, the decision to initiate replanning should be based on early and straightforward predictive factors. Only a limited number of trials have tried to identify consistent baseline clinical and dosimetric factors that are able to predict the likelihood of patients requiring ART during the course of their treatment. Pre‐treatment predictors published in the literature are (1) higher initial mean PG dose,^18^, ^61^, ^81^ (2) higher initial weight,^19^ (3) higher BMI,^61^ (4) larger CTV,^19^ (5) higher T stage,^25^, ^61^ (6) higher N disease,^19^, ^61^ (7) initial small volume of the PG,^61^ (8) overlap of the volume of the PG and TVs,^61^ (9) the administration of concomitant chemoradiotherapy,^60^, ^61^ and (10) bulkier disease.^19^, ^30^, ^81^ Concerning the latter, a high‐risk profile was defined by Brown et al. by a node volume larger than 55 mm for OPC and 15 mm for NPC.^19^ The limit for initial weight, at the time of diagnosis, was set at 100 kg, although this high threshold was not reached by patients in several trials.^60^, ^82^ Patients with NPC were proven to have a higher likelihood of requiring ART in comparison to patients with OPC.^19^, ^21^, ^25^, ^61^, ^63^, ^78^ It is important to acknowledge, however, that patients with NPC included in these trials typically presented with more advanced nodal disease (median N3 stage, in contrast to N2 stage for OPC). Lastly, Brown et al. identified viral status as a factor influencing the necessity for replanning in both OPC and NPC.^19^

Predictors occurring during treatment mentioned in literature are (1) PG volume decrease,^61^ (2) faster rate of weight loss,^18^, ^21^, ^25^, ^60^ (3) larger TV shrinkage,^21^, ^25^, ^61^ (4) greater reduction in lateral neck diameter,^25^, ^61^ (5) PG volume and density decrease,^61^ (6) change in body thickness with poorly fitting mask,^21^, ^61^ and (7) prolonged break during RT.^21^ Jensen et al. found that patients requiring ART had a median loss of 8 kg, resulting in a volume loss of 119 mL within the RT treatment field.^15^ Other studies reported weight loss of 15%–22% during RT as a threshold for replanning.^40^, ^46^, ^83^ Weppler et al. performed ART on patients with a change of body contour larger than 1.5 cm, while Hvid et al. established rigid registration couch shifts higher than 3° or 1 cm as threshold.^84^, ^85^

The mentioned trials are characterized by several limitations: a limited number of patients, various HNC subsites, a small and variable number of per‐treatment imaging sessions, and unclear criteria for the initiating of replanning, leading to a variable percentage of patients receiving ART within studies. For example, in the study of Brown et al., the primary trigger to replan was an exceeded dose to the brachial plexus, while Brouwer et al. based their decision on the dose to the PG, using an arbitrary threshold of 3 Gy.^19^, ^82^ In addition, most predictors have not been externally validated. In a validation cohort of 43 patients, Brouwer et al. confirmed the predictor initial mean parotid dose, establishing a cut‐off value of 22.2 Gy.^82^ However, the positive predictive value was only 19%, indicating that a majority of patients meeting this criterion might not profit from ART. Additionally, the PG mean dose was found to be significantly correlated with a higher nodal stage and larger nodal volumes. Conducting multivariable analyses in adequately large and appropriately selected patient cohorts is essential to untangle the various confounding factors involved.

Lastly, while per‐treatment predictors provide valuable insights by reflecting the impact of RT, their applicability is limited to adapting the RT plans for the remainder of the RT course. In contrast, pre‐treatment parameters were proven to be less effective. Although protocols for candidate selection are highly recommended, most centers still perform ad‐hoc ART without specific protocols. The POP‐ART RT trial examined the practices of 177 centers and found that while 55% of participants administered ART for head and neck cancer, only 10% of centers utilized specific online or offline protocols with predefined flagging criteria or action levels.^86^ An interesting approach was recently suggested by Gan et al., exploring a balance between pre‐ and pre‐treatment predictors, gathered as early as possible in the treatment course. A Normal Tissue Complication Probability (called “bioΔNTCP”) model was developed to select patients with a deviation in the delivered dose compared to the planned dose within the initial 2 weeks of the treatment course. The comprehensive NTCP profile consists of 180 validated models and was applied to translate planning Dmean and accumulated Dmean into nominal and “actual” NTCP values, respectively. By applying a threshold of 5% for the ΔNTCP, only a small proportion of patients required ART, sparing 73.6% and 88.2% of the patients from further ART interventions in consideration of acute and late toxicity, respectively.^87^

Commercial software systems are emerging that allow users to establish various thresholds or tolerances on DVHs metrics, commonly referred to as flagging criteria, to automatically identify patients who may benefit from replanning. However, ROs should still decide how to define the dosimetric flagging criteria in a clinically relevant way. Barragàn‐Montera et al. developed a protocol for automatically and dosimetrically triggered ART.^88^ The authors emphasize the complexity of designing universal flagging conditions to suit clinical requirements. Guidelines formulated by expert groups to establish tolerance levels are necessary to standardize flagging criteria and minimalize the existing inter‐center variability.

### 5.2 Frequency and timing of ART

Effective incorporation of ART for HNC in a clinical setting requires an optimal timing of the intervention. Presently, there is a lack of consensus on the most suitable frequency and timing for replanning in HNC patients. As shown in Tables 2 and 3, very heterogeneous timing schedules are followed in existing trials. Multiple trials tested the benefit of only one replanning during the treatment course,^20^, ^21^, ^34^, ^38^, ^57^, ^63^, ^89^ while some used regular intervals (e.g., every 10 fractions)^47^, ^54^, ^67^, ^77^, ^78^ and others followed a “maximalist” weekly planning strategy.^13^, ^15^, ^22^, ^39^ Limited studies have provided specific recommendations on the ideal schedule for replanning. Wu et al. concluded that a single replanning resulted in a reduction of the mean PG dose of 3%, and two and six replannings resulted in a 5% and 6% reduction, respectively.^64^ In a prospective study on 22 patients, Schwartz et al. demonstrated that one replanning was necessary for all patients, while a second replanning was necessary for only 36% of the patients. Furthermore, the most notable anatomical alterations seemed to manifest between the third and fourth week of the RT course.^77^ These findings were affirmed by Brown et al., stating that replanning should be considered at the start of the third week of the treatment for patients with NPC and in the fourth week for OPC patients.^90^ Zhang et al. found 3 replans in weeks 1, 2, and 5 to be comparable with 6 weekly replans for 13 OPC patients, establishing a mean reduction of the dose to the PG of 3 Gy for both approaches. Most of the advantage seemed to occur during the initial 2 weeks.^39^ Fiorentino et al. conducted a retrospective study to assess the optimal timing for ART by examining dosimetric alterations in the PGs during IMRT, by performing CBCT on treatment days 10, 15, 20, and 25. The recorded dosimetric parameters were found to be statistically significant on treatment days 10 and 15 (79). In a study involving 19 patients with NPC who underwent weekly CT scans, the most prominent dosimetric changes in TVs, PGs, brainstem, and spinal cord were observed at fractions 5 and 15.^37^ Castelli et al. advised early replanning for an optimal sparing of the PG.^22^ This advice was backed up by several trials, reporting the most significant volume and dosimetric changes on the PG to manifest in the first half of the treatment.^10^, ^11^, ^54^ Bhide et al. found a significant disparity between the true cumulative dose and the original dose after 2 weeks already.^10^ Based on these results, the consensus among most researchers is that the ideal period for replanning falls between the third and fourth week of the RT course.^16^ Additionally, efforts have been initiated to develop automated methods using machine learning to anticipate the necessity for replanning interventions, though further research is warranted.^91^

### 5.3 Practical challenges

**FIGURE 1:**
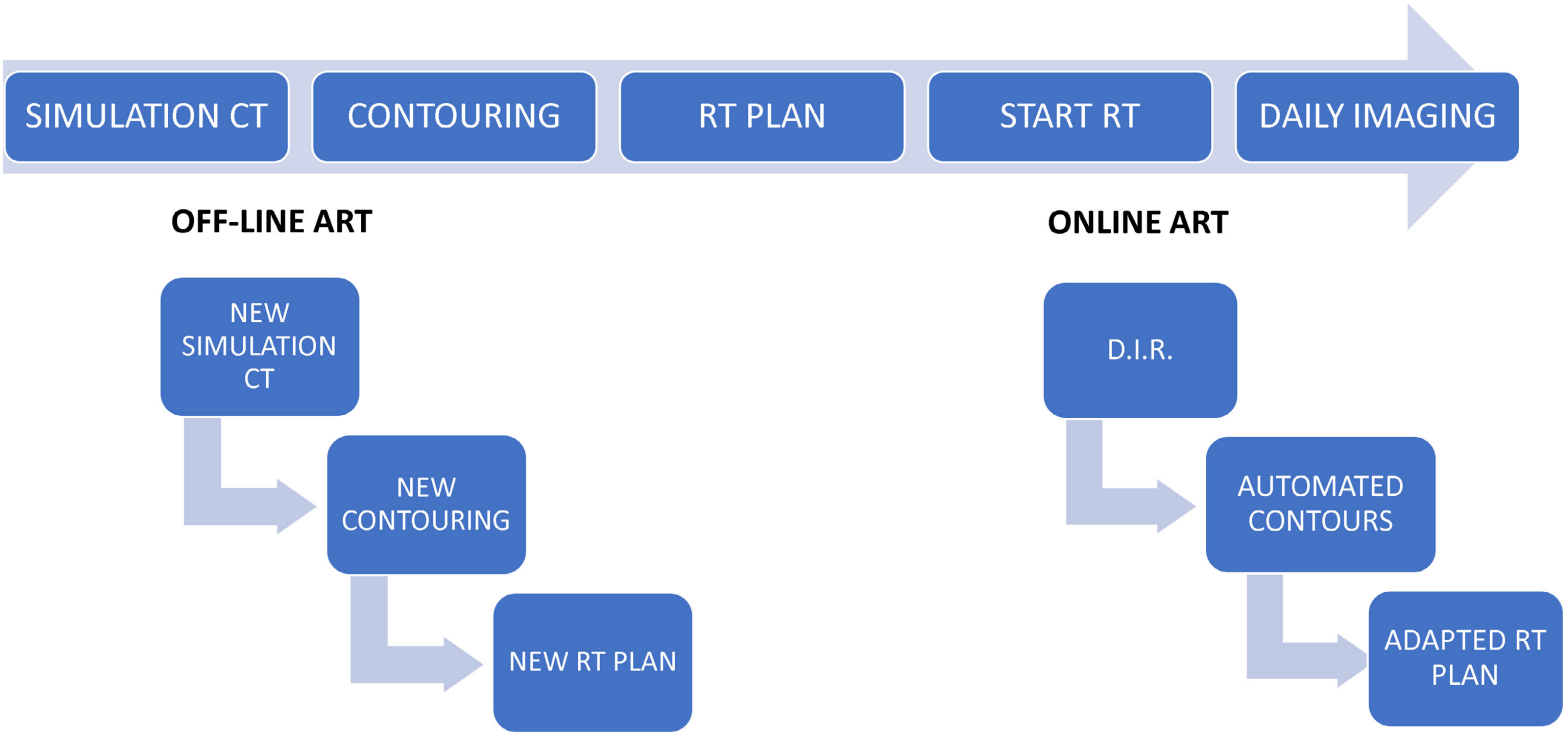
The workflow of offline and online ART. The labor‐intensive workflow of ART. In an offline setting, a re‐simulation CT is performed, on which new contouring and RT planning are executed. Commercial software for online ART is being introduced, allowing automated dose calculations and adapted planning on CBCT‐based imaging after Deformable Image Registration (D.I.R.).

The complexity of HNC RT planning and technical and logical difficulties of ART present a practical bottleneck for its broad clinical implementation. In Figure 1, the labor‐intensive workflow of ART is depicted. In an offline setting, a re‐simulation CT is performed, on which new contouring and RT planning is executed. Re‐contouring of OAR and TVs for adaptive replanning was shown to consume 2.5 h or longer.^65^, ^92^ In recent years, several companies have introduced commercial software for online ART, allowing ART to be performed with the patient on the treatment table. These software systems have been designed with the aim to streamline the ART workflow and reduce the need for human intervention, by executing automated dose calculations on new CBCT‐based imaging. New contours are derived either through the propagation of planning volumes using DIR,^93^, ^94^, ^95^, ^96^ automated delineation,^97^ or by a combination of both.^98^ In the next phase, dose‐volume histograms (DVH) are calculated on the new contours. An additional advantage of such online approach is that an adapted RT plan is proposed on the daily CBCT for every single fraction, allowing dose metrics to be followed up during the treatment through dose accumulation.^99^, ^100^, ^101^ However, these online ART software systems have their limitations. First of all, MVCT images display poorer contrast than simulation CT, which might influence the quality of the contours. Second, the current accuracy of co‐registration methods is known to be insufficient, because of the pronounced anatomical deviations that characterize HNC patients.^75^ The utilization of DIR for delineation of the PGs was demonstrated to cause an average mean dose deviation of 3.64 Gy, resulting in significant disparities in NTCP predictions for xerostomia.^102^ In addition, the automation within these software systems introduces certain “black‐box steps” that require meticulous attention. After using and testing the ETHOS technology,^88^, ^98^ Barragàn‐Montero et al. stated that, ideally, an experienced RO should perform a visual inspection of all contours.^88^ These limitations may explain why in the existing trials, summarized in Table 2, off‐line ART approaches have shown more consistent dose benefits. Lastly, in the current version of ETHOS, the user can only choose at the planning stage to use an adaptive workflow, with no possibility to change that decision during RT. The mentioned issues with online ART thus reintroduce similar challenges as those associated with the concept of off‐line ART, and the benefits of both off‐line and online ART should still be weighed against the extension of treatment time. In the first prospective CBCT‐based online ART trial, Avkshtol et al. concluded that, given the time investment, patient‐specific adaptation questions should be answered.^103^

### 5.4 Adaptation of target volumes

Considering the current unreliability of both image registration and automated re‐contouring, TV delineation should, in any circumstances, be carefully checked by the RO, to prevent recurrences due to inadequately reduced CTV. Regarding TV re‐delineation, another pressing question arises. For patients in which a reduction of the primary tumor volume is objectified, what specific adjustments should be made to the borders of the high‐ and intermediate‐dose CTV? Most studies in which replanning is performed do not state the specific procedure that was followed to account for this problem. For example, in the largest prospective trial on clinical outcomes after ART, the authors did not specify whether the initial GTV was consistently encompassed in the replan CTVs.^78^ Gensheimer et al. drew an analogy to the context of induction chemotherapy preceding RT.^7^ While clinical practice guidelines advise contouring both the GTV and high‐dose CTV based on the pre‐chemotherapy imaging,^104^ studies have demonstrated equivalent recurrence rates when delineation was performed mainly using post‐chemotherapy imaging, under the condition that the pre‐chemotherapy GTV area was incorporated in the high‐dose CTV.^105^ Consequently, Gensheimer et al. concluded that, given the lack of definitive data, it would be reasonable for adaptive replanning to include the pre‐treatment volume in either the high‐ or intermediate‐dose CTV, leaving the decision to the discretion of the responsible RO. However, inter‐observer variability (IOV) for the delineation of OAR and TVs is a well‐established issue for HNC patients.^106^ A significantly higher IOV among centers was found in the adaptive setting compared to pre‐treatment delineation.^107^ This finding was explained by the lack of imaging information from PET/CT or MRI in the adaptive setting. However, the effect of these imaging modalities on IOV has been debated with conflicting results among studies.^108^, ^109^ In addition, mid‐treatment imaging is responsive to changes induced by treatment, such as heightened perfusion, adding complexity to its interpretation. Consequently, in addition to the absence of alternative imaging modalities for the re‐delineation during RT, the variation in institutional adaptation practice likely contributes to the observed increased IOV in the adaptive setting. Consensus guidelines for the adaptation of TVs during treatment are thus urgently needed.

Furthermore, it is well known that one of the potentials of daily ART is to enable a margin reduction from CTV to PTV.^110^, ^111^ Van Kranen et al. reported that a margin reduction from 5 to 0 mm, following a CBCT‐based adaptive strategy, led to a reduction of Dmean of 1 Gy/mm on several OAR. However, since target coverage was compromised in a significant subset of patients, the authors advocate for well‐founded ART protocols.^112^ The DARTBOARD clinical trial will study daily ART on HNC patients using an AI‐driven algorithm, applying PTVs of only 1 mm for intra‐fractional motion (NCT04883281).

## 6 FUTURE PERSPECTIVES

As technology progresses, with more accurate automated contouring, better in‐room imaging, and faster plan optimization software, is it likely that the factors currently hindering clinical integration of ART will be overcome. Deep learning for automated segmentation for both OAR and TV shows promising evolutions on several imaging modalities. In addition, knowledge‐based planning algorithms have been developed that utilize pre‐approved RT plans as a foundation of the IMRT optimizer.^113^, ^114^, ^115^ Currently, planning CT remains the standard imaging for routine RT in the majority of institutions. The recent introduction of MR‐Linac technology, which integrates a linear accelerator with a 1.5 Tesla MRI, holds the potential to facilitate MR‐guided ART workflows, through daily online MRI.^116^, ^117^ In addition to enhanced soft‐tissue contrast, another benefit of MR‐guided ART is the capability to monitor tumor response throughout treatment, forming the basis for response‐adapted RT. In contrast to the conventional anatomy‐adapted RT, where the adapted RT replan reproduces the original TVs and doses adjusted to the new anatomy, response‐adapted ART involves altering the TVs and/or RT doses based on the response to RT, objectified by functional imaging techniques including PET and MRI. Particularly, diffusion‐weighted MRI shows promise as a predictive imaging biomarker in HNC patients, although findings remain inconsistent depending on the analyzed parameters.^118^, ^119^, ^120^ Whether dose escalation is a viable approach to enhance LRC in HNC is still a question under debate, but preliminary reports suggest that this concept is feasible without an increased risk of toxicity.^58^, ^121^, ^122^ Prospective randomized trials evaluating the oncologic and toxicity outcomes of PET/CT‐based ART have not been able to show a benefit,^123^, ^124^ while a randomized trial on 81 patients receiving an MRI‐based RT boost showed improved LRC without reaching its primary end‐point of disease‐free survival.^125^ The results of several studies are still awaited (NCT03224000, NCT03972072, NCT04242459, and NCT03935672). Mid‐treatment FDG/PET imaging can also be used to select patients for de‐escalated radiotherapy in good prognosis HPV+ OPC, as shown by Allen et al.^126^ Finally, facilitation of ART is crucial for the utilization of proton therapy in clinical practice for HNC patients. Proton therapy has been proven a promising treatment for patients with HNC, since it offers the ability to deliver high doses to the TV while minimizing the dose to the adjacent OAR.^6^ As the energy deposition of proton particles takes place within a narrow range of depth in tissue, the significance of adaptation to anatomical changes is even more pronounced. There has been growing interest in clinically integrating an online daily adaptive proton therapy (DAPT) workflow to effectively address inter‐fractional changes.^127^, ^128^ The various steps necessary prior to the introduction of DAPT were discussed by Albertini et al.^129^

## 7 CONCLUSION

Although based on non‐randomized and mostly retrospective trials, there is accumulating evidence that ART holds the potential to improve normal tissue constraints and target coverage in HNC patients with extensive anatomical changes during treatment. However, randomized evidence is not yet available to confirm clinical benefits regarding toxicity and tumor control. Furthermore, several questions remain regarding accurate patient selection, the ideal frequency and timing of replanning, and the appropriate way for image registration and dose calculation. There is an urgent need for well‐designed randomized prospective trials, with a predetermined protocol for both image registration and dose summation, for further exploration of the dosimetric and clinical benefits of ART. A true ART strategy should likely be personalized to each patient depending on his/her risk profile, for which improved understanding of influential pretreatment factors is essential. Considering the current labor intensity and cost of the approach, continued research on automation of the adaptive process should be encouraged.

## AUTHOR CONTRIBUTIONS

**Sandra Nuyts:** Conceptualization (lead); investigation (equal); methodology (equal); writing – original draft (equal); writing – review and editing (equal). **Heleen Bollen:** Conceptualization (equal); investigation (equal); writing – original draft (equal); writing – review and editing (equal). **Avrahram Eisbruch:** Writing – review and editing (supporting). **Primoz Strojan:** Writing – review and editing (supporting). **William M. Mendenhall:** Writing – review and editing (supporting). **Sweet Ping Ng:** Writing – review and editing (supporting). **Alfio Ferlito:** Writing – review and editing (supporting).

## Data Availability

Data sharing is not applicable to this article as no new data were created or analyzed in this study.
